# Evaluating Nanoshells and a Potent Biladiene Photosensitizer for Dual Photothermal and Photodynamic Therapy of Triple Negative Breast Cancer Cells

**DOI:** 10.3390/nano8090658

**Published:** 2018-08-25

**Authors:** Rachel S. Riley, Rachel K. O’Sullivan, Andrea M. Potocny, Joel Rosenthal, Emily S. Day

**Affiliations:** 1 Department of Biomedical Engineering, University of Delaware, Newark, DE 19716, USA; rsriley@seas.upenn.edu (R.S.R.); rkos@udel.edu (R.K.O.); 2 Department of Chemistry & Biochemistry, University of Delaware, Newark, DE 19716, USA; apotocny@udel.edu; 3 Department of Materials Science & Engineering, University of Delaware, Newark, DE 19716, USA; 4 Helen F. Graham Cancer Center & Research Institute, Newark, DE 19713, USA

**Keywords:** photothermal therapy, photodynamic therapy, nanoparticle, biladiene, palladium, photosensitizer, apoptosis, synergy, photoresponsive, cancer

## Abstract

Light-activated therapies are ideal for treating cancer because they are non-invasive and highly specific to the area of light application. Photothermal therapy (PTT) and photodynamic therapy (PDT) are two types of light-activated therapies that show great promise for treating solid tumors. In PTT, nanoparticles embedded within tumors emit heat in response to laser light that induces cancer cell death. In PDT, photosensitizers introduced to the diseased tissue transfer the absorbed light energy to nearby ground state molecular oxygen to produce singlet oxygen, which is a potent reactive oxygen species (ROS) that is toxic to cancer cells. Although PTT and PDT have been extensively evaluated as independent therapeutic strategies, they each face limitations that hinder their overall success. To overcome these limitations, we evaluated a dual PTT/PDT strategy for treatment of triple negative breast cancer (TNBC) cells mediated by a powerful combination of silica core/gold shell nanoshells (NSs) and palladium 10,10-dimethyl-5,15-bis(pentafluorophenyl)biladiene-based (Pd[DMBil1]-PEG_750_) photosensitizers (PSs), which enable PTT and PDT, respectively. We found that dual therapy works synergistically to induce more cell death than either therapy alone. Further, we determined that low doses of light can be applied in this approach to primarily induce apoptotic cell death, which is vastly preferred over necrotic cell death. Together, our results show that dual PTT/PDT using silica core/gold shell NSs and Pd[DMBil1]-PEG_750_ PSs is a comprehensive therapeutic strategy to non-invasively induce apoptotic cancer cell death.

## 1. Introduction

Light-activated therapies offer less invasive, more precise, and safer alternatives for cancer treatment than conventional therapies such as chemotherapy, surgery, and radiation. In light-activated therapies, photoresponsive materials are intravenously injected into the body and accumulate within tumor tissue via the enhanced permeability and retention (EPR) effect, whereby the administered materials achieve high intratumoral concentrations due to the leaky vasculature and poorly organized lymphatic system characteristic of tumors [1,2]. Once the agents reach the tumor site, light is applied to activate the treatment and to harm the surrounding cancer cells. Due to the benefits afforded by light-activated therapies, photoresponsive materials have been developed to elicit a number of therapeutic effects including drug and gene delivery, hyperthermia, or the production of reactive oxygen species or other cytotoxic radicals [3,4,5,6,7]. However, the success of light-activated therapies depends on the sufficient intratumoral accumulation of the photoresponsive materials (while maintaining minimal off-target dark toxicity), as well as on tumor physiology. A promising strategy to ensure complete tumor eradication would be to combine different types of light-activated therapies to harness the multiple discrete benefits associated with the use of light for cancer treatment while minimizing the limitations of any individual therapy.

In this study, we evaluated a powerful combination of photothermal and photodynamic therapies (PTT and PDT, respectively) to irreversibly kill cancer cells by overcoming the limitations of each therapy alone. In PTT, nanoparticles (NPs) embedded within tumors produce heat upon application of laser light tuned to their peak plasmon resonance wavelength [3,8]. The heat generated is sufficient to cause tumor cell death in the areas immediately surrounding the activated NPs. The main consideration for utilizing PTT as an independent treatment strategy is that it can cause necrotic cell death if the treatment parameters are not carefully controlled, and PTT-induced necrosis has recently been correlated with local inflammation and disease recurrence [9,10]. It is more desirable to control the laser exposure conditions and NP doses to induce apoptosis, which does not produce detrimental outcomes [9,10]. We hypothesized that combining PTT with PDT would enable the use of much lower laser powers and NP concentrations to primarily induce apoptotic, rather than necrotic, cell death from PTT. 

In PDT, photosensitizers (PSs) in tumor tissue absorb externally applied light and transfer the energy to ground state molecular oxygen to produce singlet oxygen (^1^O_2_), which is toxic to cells and ultimately induces localized cell death within the area of irradiation [11,12,13]. PDT is a promising treatment strategy for certain types of cancers and skin conditions [14] because it is less invasive than surgical options [15], has fewer side effects than radiation or chemotherapy [13,16], and has been shown to stimulate antitumor immune responses [17,18]. Despite these promising benefits of PDT, its widespread clinical use is met with three key limitations. First, PSs often have high toxicity to healthy tissues even without light application, which limits the allowable administered dosages [19]. Further, successful PDT requires sufficient oxygen presence in the native tissue to produce toxic ^1^O_2_. However, the microenvironment deep within solid tumors is often hypoxic, thereby rendering the PSs ineffective in these regions [19]. Lastly, most PSs for PDT are activated by short wavelengths of light (<600 nm) that cannot penetrate tissue, resulting in uneven therapeutic effects throughout the tumor space [13,19]. Researchers have developed PSs that can be activated with longer wavelengths of light for enhanced tissue penetration, but unfortunately this approach is still ineffective in hypoxic tumor regions [20,21], and these PSs still suffer from relatively high off-target toxicities even without light application. 

**Figure 1:**
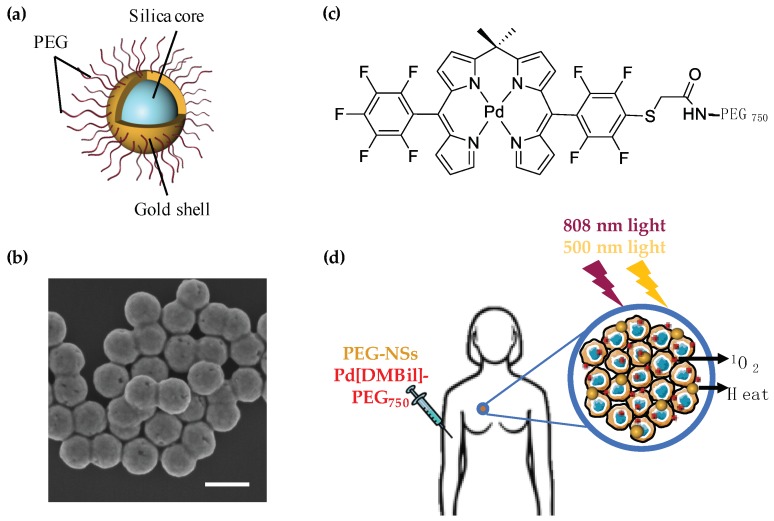
(**a**) Schematic of poly(ethylene glycol) nanoshells (PEG-NSs) used to induce photothermal therapy (PTT); (**b**) Scanning electron micrograph showing the size and monodispersity of NS. Scale = 200 nm; (**c**) Chemical structure of Pd[DMBil1]-PEG_750_ photosensitizers used to mediate photodynamic therapy (PDT); (**d**) Scheme showing the proposed therapeutic strategy of dual PTT/PDT to treat triple negative breast cancer (TNBC). First, PEG-NSs and Pd[DMBil1]-PEG_750_ would be intravenously injected and accumulate within solid tumors based on the enhanced permeability and retention (EPR) effect. Then, an 808 nm continuous wave laser and 500 nm light would be applied to activate PEG-NSs and/or Pd[DMBil1]-PEG_750_, respectively, to produce heat and singlet oxygen that is toxic to the surrounding cancer cells.

In this work, we used nanoshells (NSs) composed of silica cores and thin gold shells (~150 nm in diameter, Figure 1a,b) that were coated with poly(ethylene glycol) (PEG) as the photoresponsive material for PTT and PEGylated palladium 10,10-dimethyl-5,15-bis(pentafluorophenyl)biladiene (Pd[DMBil1]-PEG_750,_ Figure 1c) as the photosensitizer for PDT. We selected NSs as the mediator of PTT because they maximally absorb near infrared (NIR) light that can safely penetrate several centimeters of healthy tissue to reach NSs embedded within tumors [3,22,23,24]. Further, NSs have impressive safety profiles in both human trials and in animal models, and they are currently being investigated in clinical trials to mediate PTT in several types of tumors [25,26,27]. Another benefit afforded by gold-based NPs, such as NSs, is that simple gold-thiol conjugation chemistry can be used to attach passivating agents, such as PEG, or targeting agents to their surfaces. For this work, we coated NSs with PEG (PEG-NSs) for increased stability. To mediate PDT, we used a novel PS recently developed in our lab, Pd[DMBil1]-PEG_750_, that is soluble in aqueous solutions, absorbs strongly out to ~575 nm, and efficiently produces singlet oxygen [28]. Our initial investigations using Pd[DMBil1]-PEG_750_ for PDT demonstrated that this PS is inherently nontoxic in the dark and highly effective for inducing cell death upon light activation, with an incredible phototoxicity index of ~5300 [28]. Due to the limitations of PTT and PDT as individual treatment strategies explained above, we anticipated that dual PTT/PDT using PEG-NSs and Pd[DMBil1]-PEG_750_, respectively, would overcome the limitations of each therapy to provide a more comprehensive solution to treat solid tumor cancers (Figure 1d).

Our hypothesis that combining PTT and PDT will synergistically improve the efficacy of each treatment stems from previous studies that evaluated other photoresponsive materials for dual therapies [29,30,31,32,33]. For example, NIR-absorbing NPs have been used as carriers for PSs tethered to their surfaces, which allows these conjugates to mediate PDT and PTT simultaneously [33,34]. Alternatively, our approach is to induce dual PTT/PDT with the two separate agents mixed together, but not physically attached to each other, with the anticipated benefit that both can distribute throughout the tumor independently to mediate each therapy, increasing the likelihood that all regions of the tumor would receive PTT and/or PDT. Further, in this technique, the output powers of each light source can be easily controlled to maximize therapeutic outcomes. We evaluate this therapeutic strategy in triple negative breast cancer (TNBC) cells because this disease is characterized by solid tumors that proliferate quickly, and thus it provides a model to study phototherapies for solid tumors [35]. First, we show that both PEG-NSs and Pd[DMBil1]-PEG_750_ are stable in aqueous solutions and that they retain their individual properties to enable PTT or PDT, respectively. Further, we show that PTT and PDT work synergistically to induce more cell death than either therapy alone. Finally, we demonstrate that dual PTT/PDT primarily induces apoptotic cell death over necrosis at low light dosages. Together, these results indicate that dual PTT/PDT using PEG-NSs and Pd[DMBil1]-PEG_750_ PSs is a promising strategy to treat TNBC and other solid tumor cancers, and it provides a critical foundation for future in vivo studies towards the goal of clinical translation.

## 2. Materials and Methods 

### 2.1. Nanoshell Synthesis and Functionalization

NSs were synthesized according to published methods [36]. Briefly, colloidal gold NPs formed by the Duff et al. method [37] were combined with 120 nm aminated silica NPs (Nanocomposix, San Diego, CA, USA) and reacted for several days to form “seed.” The seed solution was centrifuged (2000× *g*, 20 min, twice) and the supernatant containing unreacted gold colloid was removed. The purified seed, which was diluted in water, was reacted with potassium carbonate containing HAuCl_4_ (Sigma Aldrich, St. Louis, MO, USA) and formaldehyde to create NSs, which were then purified by centrifugation (1500× *g*, 5 min, twice). Next, NSs were combined with 5 kDa methoxy poly(ethylene) glycol with a thiol end group (mPEG-SH, Laysan Bio, Arab, AL, USA) to a final mPEG-SH concentration of 5 mM, and the solution was reacted overnight at 4 **°**C to form PEG-NSs. Lastly, PEG-NSs were purified by centrifugation (1500× *g*, 7 min, thrice) and stored in ultrapure water at 4 **°**C until use. 

### 2.2. Pd[DMBil1]-PEG_750_ Synthesis

Pd[DMBil1]-PEG_750_ was synthesized using methods described previously [28,38,39,40,41]. Reactions requiring an inert atmosphere were carried out in oven-dried round bottomed flasks fitted with Suba-seal rubber septa purchased from Chemglass and maintained under positive nitrogen pressure using standard schlenk techniques. Reagents and solvents were purchased from Strem (Newburyport, MA, USA), VWR (Radnor, PA, USA), Sigma-Aldrich (St. Louis, MO, USA), Fisher (Hampton, NH, USA), Matrix Scientific (Columbia, SC, USA), Acros (NJ, USA), Decon Laboratories Inc. (King of Prussia, PA, USA), Cambridge Isotopes Laboratories (Tewksbury, MA, USA), or Alfa Aesar (Haverville, MA, USA). All solvents used for synthesis were of reagent grade or better, and anhydrous solvents were dried via passage through activated alumina [41]. Column chromatography was carried out with 40–63 μm silica gel purchased from Silicycle, or C_2_-silica prepared as detailed in a published procedure [28]. Precoated glass plates from Silicycle were used for thin layer chromatography (TLC) and, when necessary, visualization was aided by UV light. The title compound was characterized by a combination of ^1^H, ^13^C, and ^19^F NMR spectroscopy, as well as by high-resolution mass spectrometry. Analytical data matched that previously reported for this compound [28].

### 2.3. Characterization and Stability of PEG-NSs and Pd[DMBil1]-PEG_750_

PEG-NSs at 5.5 × 10^9^ NS/mL (corresponding to optical density (OD) 2) and/or 10 μM Pd[DMBil1]-PEG_750_ diluted in complete cell culture medium (Dulbecco’s Modified Eagle Medium (DMEM) supplemented with 10% fetal bovine serum and 1% penicillin-streptomycin) were characterized by UV-visible spectroscopy (Cary60 spectrometer, Agilent, Santa Clara, CA, USA) before and after a 24 h incubation at 37 **°**C with 5% CO_2_ to demonstrate their stability. Samples were scanned from 400–1100 nm using a scan rate of 2400 nm/s, and a baseline of media only was subtracted from the scan. Samples were also characterized by dynamic light scattering (DLS) and zeta potential (Litesizer500, AntonPaar, Graz, Austria), and the reported hydrodynamic diameter is the z-average mean from three sample measurements. PEG-NS samples for scanning electron microscopy (SEM) were diluted to 2.7 × 10^9^ NS/mL (OD 1) in 200 proof ethanol and were dried directly onto SEM sample holders prior to imaging (S4700, Hitachi, Chiyoda, Tokyo, Japan). 

### 2.4. Temperature and ^1^O_2_ Production during PTT and PDT

Solutions of PEG-NSs (5.5 × 10^9^ PEG-NSs/mL) and/or Pd[DMBil1]-PEG_750_ (4 μM) were diluted in ultrapure water and placed into black-walled 96-well plates for ^1^O_2_ analysis or into 24-well plates for temperature analysis. For temperature analysis, samples were irradiated for 3 min with a continuous wave 808 nm laser (B&W Tek) at 0.75 W/cm^2^, which activates PEG-NSs for PTT, and/or λ_exc_ > 500 nm light for 10 min (Artograph LightPad 930 light plate with a 500 nm long pass filter, which activates PSs for PDT; Delano, MN, USA). Temperature measurements were recorded using an FLIR A5 thermal camera (Wilsonville, OR, USA). For ^1^O_2_ detection, 10 μL of a 0.5 mM stock solution of singlet oxygen sensor green (SOSG) was added to 90 μL of sample containing PEG-NSs and/or Pd[DMBil1]-PEG_750_. The plates were irradiated with the LightPad for 10 min and then SOSG fluorescence was read on a Synergy H1 plate reader (BioTek, Winooski, VT, USA). Irradiation experiments were repeated four times to generate singlet oxygen production curves.

### 2.5. Cell Culture

MDA-MB-231 TNBC cells were purchased from American Type Culture Collection (ATCC, Manassas, VA, USA) and cultured in DMEM supplemented with 10% fetal bovine serum and 1% penicillin-streptomycin. Cells were cultured in T75 cell culture flasks and incubated at 37 °C in a 5% CO_2_ humidified environment. Cells were passed between flasks or into sample plates by detaching the cells from the flasks with Trypsin-EDTA when they reached 80–90% confluency and were counted with a hemocytometer prior to transferring to a new flask or well plate.

### 2.6. Cellular Binding and Uptake of Pd[DMBil1]-PEG_750_ and PEG-NSs

To analyze cellular binding and uptake of Pd[DMBil1]-PEG_750_ and PEG-NSs by fluorescence or darkfield imaging, cells were plated at 15,000 or 25,000 cells/well, respectively, in glass bottom 8-well plates with removable well chambers, and were incubated overnight. Then, 2.7 × 10^9^ PEG-NSs/mL and/or 1 mM Pd[DMBil1]-PEG_750_ diluted in complete cell culture medium was added to cells for 24 h protected from light. Cells were fixed with 4% formaldehyde for 15 min and rinsed three times with 1X PBS. Cells were then stained with phalloidan (Cell Signaling Technology, Danvers, MA, USA) overnight at 4 **°**C to visualize F-actin on the cell cytoskeleton. Well chambers were removed and slides were mounted with DAPI-containing mounting media to label cell nuclei (Vectashield, Burlingame, CA, USA). Cells were imaged with a Zeiss Axioobserver Z1 Inverted Fluorescent Microscope (Oberkochen, Germany) using the FITC (F-actin), DsRed (Pd[DMBil1]-PEG_750_), and DAPI (nuclei) fluorescence channels and a darkfield condenser (to visualize scattering from PEG-NSs). 

### 2.7. Assessment of Cells’ Metabolic Activity Following PTT and/or PDT

Cells were treated with 0 or 5.5 × 10^9^ PEG-NSs/mL and/or 0 or 0.25 μM Pd[DMBil1]-PEG_750_ overnight and were then irradiated for 2 min/well with the 808 nm continuous wave laser at a power density of 0.75 W/cm^2^ and/or 10 min with the light plate. After overnight incubation, the media was removed and an Alamar blue viability reagent (ThermoFisher, Waltham, MA, USA; diluted 1:10 in complete cell culture media) was added per manufacturer recommendations. Sample fluorescence was measured on the plate reader with excitation and emission wavelengths of 560 nm and 590 nm, respectively. To analyze the data, background (Alamar blue reagent without cells) was subtracted from the fluorescence reading in each well. Fluorescent signal from triplicate wells was averaged and normalized to untreated cells that were not exposed to light, PEG-NSs, or Pd[DMBil1]-PEG_750_. Data shown is from at least three experiments that were each run with triplicate wells. The additive or synergistic effects of the treatments were calculated using a coefficient of drug interaction (CDI) from cells treated with PEG-NSs and Pd[DMBil1]-PEG_750_ and each light source according to the equation $\mathrm{CDI}=\frac{\mathrm{AB}}{\left(A\times B\right)}$ [42,43]. In this equation, *AB* is the viability of cells treated with both light sources (i.e., dual PTT/PDT), and *A* and *B* are cell viability following treatment with either light source (i.e., PTT or PDT alone). The PTT/PDT experimental data with NSs and Pd[DMBil1]-PEG_750_ was analyzed by 1-way ANOVA with post hoc Tukey. 

### 2.8. Analysis of the Mechanisms of Cell Death Induced by PTT and/or PDT

For dual PTT/PDT experiments, cells were treated with NSs diluted to 0 or 5.5 × 10^9^ NS/mL and/or 0 or 4 μM Pd[DMBil1]-PEG_750_ overnight and were then irradiated for 20 min with the light plate or for 2 min/well with the 808 nm laser at 0.85 W/cm^2^. After incubating cells for 1 h, an AnnexinV-FITC stain (Cayman Chemicals, Ann Arbor, MI, USA) was conducted via manufacturer instructions. Briefly, cells were lifted with trypsin, washed once with 1× binding buffer (300× *g*, 5 min), and resuspended in 50 μL binding buffer containing 1:500 AnnexinV-FITC and 1:2000 propidium iodide (PI) stains for 10 min protected from light. The samples were then diluted with 150 μL 1× binding buffer and run on an Acea Novocyte 2060 flow cytometer with the FITC (excitation, 488 nm; emission, 530/30 nm) and PerCP (excitation, 488 nm; emission, 675/30 nm) channels. Data analysis was performed in NovoExpress software (ACEA Biosciences, San Diego, CA, USA), and positive stained gates were based off of unstained cells. Single stained controls were used for compensation. Data shown are averaged amongst three independent experiments.

## 3. Results

### 3.1. PEG-NSs and Pd[DMBil1]-PEG_750_ Are Stable and Retain Their Individual Photophysical Properties When Combined

**Figure 2:**
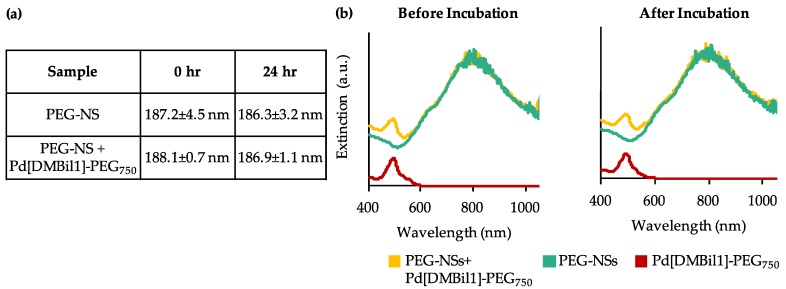
(**a**) Hydrodynamic diameter (z-average) and (**b**) Extinction spectra of PEG-NSs and/or Pd[DMBil1]-PEG_750_ in cell culture medium before (**left**) and after (**right**) a 24 h incubation period.

Bare NSs were first characterized by SEM (Figure 1b), which showed that NSs are highly monodisperse and approximately 150 nm in diameter prior to PEGylation. Zeta potential measurements of PEG-NSs diluted in water revealed they have a surface charge of −26.6 ± 0.9 mV. The stability of PEG-NSs and Pd[DMBil1]-PEG_750_ was examined by diluting each to final concentrations of 5.5 × 10^9^ NS/mL and 10 μM, respectively, in complete cell culture medium and measuring the hydrodynamic diameter and extinction spectrum immediately and then again following a 24 h incubation period at 37 °C. The original hydrodynamic diameter of PEG-NSs prior to incubation was 187.2 ± 4.5 nm (Figure 2a). The size of PEG-NSs did not change following the 24 h incubation, indicating that PEG-NSs are stable in cell culture media (Figure 2a). Further, the hydrodynamic diameter of PEG-NSs remained the same even with Pd[DMBil1]-PEG_750_ combined into the solution, demonstrating that PEG-NSs are not complexed with the photosensitizers (Figure 2a). These results were confirmed by analyzing the extinction spectrum using UV visible spectroscopy (Figure 2b). The key extinction features for NSs include a narrow peak around 800 nm, a small shoulder at ~650 nm, and a 3:1 peak:trough ratio, which indicates that the NSs have complete gold shells and are not aggregating in solution. The absorbance spectrum for Pd[DMBil1]-PEG_750_ is characterized by a narrow peak at ~500 nm and a secondary peak at ~550 nm [28]. The spectra of PEG-NSs and Pd[DMBil1]-PEG_750_ were nearly identical before and after the 24 h incubation, confirming that both are stable in cell culture medium (Figure 2b). We also combined PEG-NSs and Pd[DMBil1]-PEG_750_ into a single solution to ensure that both photoresponsive materials remain stable and independent. This was important because dual PTT/PDT requires that each agent retain its individual optical properties to be effective. We found that all of the key optical characteristics of both PEG-NSs and Pd[DMBil1]-PEG_750_ were retained following the 24 h incubation. Together, these results demonstrate that PEG-NSs and Pd[DMBil1]-PEG_750_ are stable in cell culture medium, and that each material retains its individual photophysical properties to mediate PTT or PDT, respectively. 

### 3.2. PEG-NSs and Pd[DMBil1]-PEG_750_ Produce Heat and ^1^O_2_ When Combined

**Figure 3:**
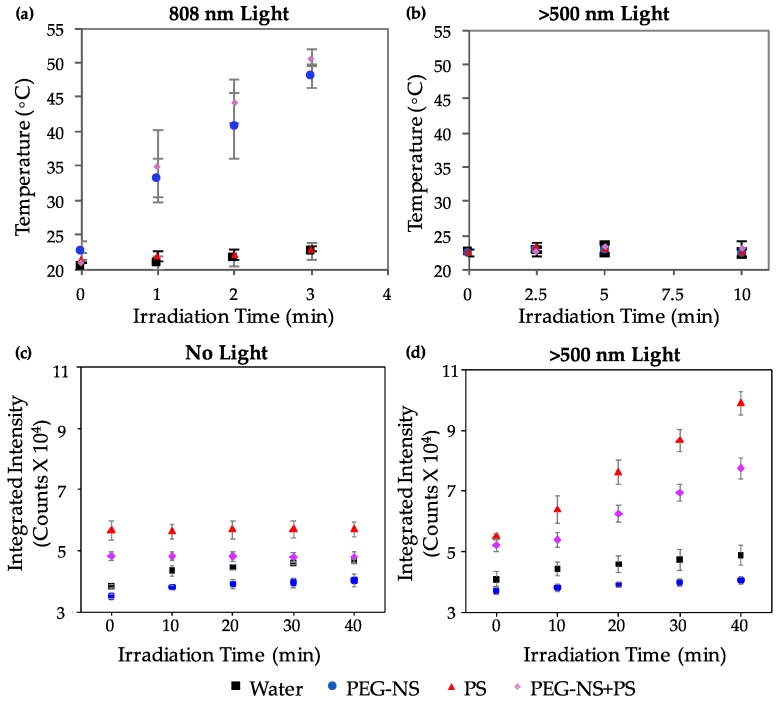
(**a**) Temperature measurements of water, aqueous solutions of PEG-NSs, aqueous solutions of Pd[DMBil1]-PEG_750_ PSs, or aqueous solutions of both PEG-NSs + PSs during irradiation with an 808 nm continuous wave (CW) laser for 3 min or (**b**) With a λ_exc_ > 500 nm light plate for 10 min; (**c**) Integrated intensity of fluorescence measurements from the singlet oxygen sensor green probe in solution with water, aqueous solutions of PEG-NSs, aqueous solutions of Pd[DMBil1]-PEG_750_ PSs, or aqueous solutions of both PEG-NSs + PSs in the dark or (**d**) With a λ_exc_ > 500 nm light plate for four 10 min periods of irradiation.

To evaluate the PTT and PDT efficiencies of each photoresponsive material, we irradiated solutions of 5.5 × 10^9^ PEG-NSs/mL and/or 4 μM Pd[DMBil1]-PEG_750_ with the 808 nm laser or the λ_exc_ > 500 nm light plate, and evaluated both the temperature and singlet oxygen production under each irradiation condition. The temperature measurement data (Figure 3a,b) show that heat is generated when solutions containing PEG-NSs are irradiated with the 808 nm laser (Figure 3a). Further, no temperature increases are observed from solutions containing Pd[DMBil1]-PEG_750_, PEG-NSs, or both after irradiation with the light plate (Figure 3b). This demonstrates that temperature increases are only a result of PEG-NSs combined with the 808 nm continuous wave laser.

For singlet oxygen measurements, each irradiation was repeated 4 times to generate curves showing increasing singlet oxygen generation from each repeated dose of light exposure (Figure 3c,d). These data show that only the combination of the λ_exc_ > 500 nm light and Pd[DMBil1]-PEG_750_ results in ^1^O_2_ production for PDT-mediated cell death. Additionally, no ^1^O_2_ was produced from 808 nm laser irradiations, demonstrating that any effect of PDT results solely from PS exposure to the >500 nm light plate. Together, these results demonstrate that combining PEG-NSs and Pd[DMBil1]-PEG_750_ does not change the photophysical properties of either material, and confirms that they can be used together for PTT and PDT, respectively. Importantly, the amount of heat and ^1^O_2_ produced from the mixture of both materials were similar to that of each individual agent alone. This similarity suggests that any cell death from PDT is due to the PSs (i.e., Pd[DMBil1]-PEG_750_) and >500 nm light treatment, and that any PTT effect is mediated by PEG-NSs and 808 nm light.

### 3.3. PEG-NSs and Pd[DMBil1]-PEG_750_ Are Internalized within Cells

**Figure 4:**
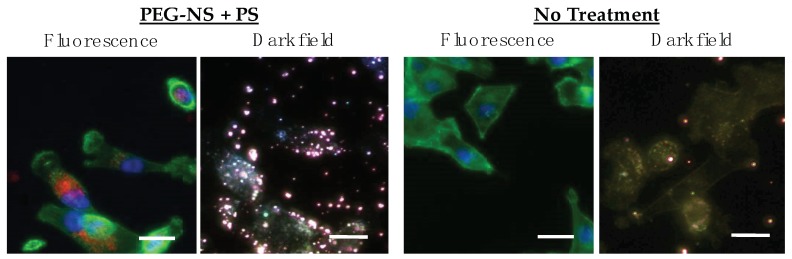
Fluorescence and darkfield images showing the binding and uptake of the Pd[DMBil1]-PEG_750_ PSs and PEG-NSs, respectively, by MDA-MB-231 TNBC cells following a 24 h incubation period. Nuclei are blue (DAPI), F-actin in the cytoskeleton is green (phalloidan), and Pd[DMBil1]-PEG_750_ is red. In darkfield microscopy, PEG-NSs are visualized as bright gold spots. Scale = 25 μm.

Next, we examined cellular uptake of PEG-NSs and Pd[DMBil1]-PEG_750_ in TNBC cells. To do this, cells were treated with 2.7 × 10^9^ PEG-NSs/mL and/or 1 mM Pd[DMBil1]-PEG_750_ for 24 h, fixed, and cytoskeletons were stained with Phalloidan. Fluorescence imaging confirmed that Pd[DMBil1]-PEG_750_ is internalized by cells during the incubation period, as indicated by the red fluorescent signal, and darkfield microscopy revealed that PEG-NSs (gold spots) are bound to or internalized by cells as well (Figure 4). Darkfield imaging of cells treated with no PEG-NSs or Pd[DMBil1]-PEG_750_ show minimal background signal that may be attributed to dust, but the scattering signal from cells treated with PEG-NSs is substantially stronger. These results were encouraging given that both agents need to be in close proximity to cells or internalized within cells to be effective for PTT and PDT, leading us to investigate their use for dual therapy as a strategy to treat TNBC. 

### 3.4. Dual PTT/PDT Is a Potent Strategy to Treat TNBC

**Figure 5:**
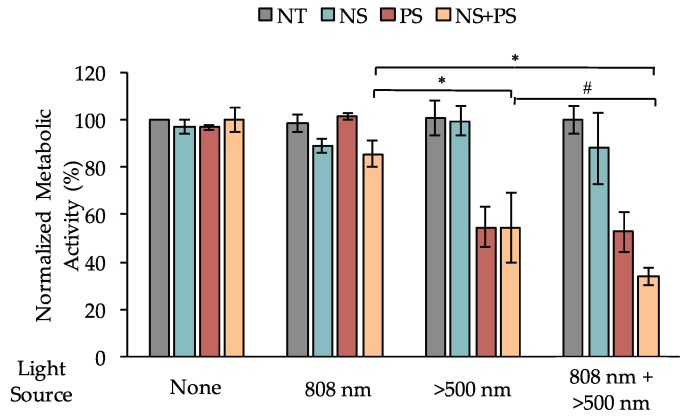
Normalized metabolic activity of MDA-MB-231 TNBC cells following treatment with PEG-NSs and/or the Pd[DMBil1]-PEG_750_ PSs for 24 h followed by irradiation with 808 nm and/or λ_exc_ > 500 nm light sources. # *p* = 0.06 and * *p* < 0.05 by 1-way ANOVA with post hoc Tukey.

To evaluate dual PTT/PDT as a strategy to treat TNBC, we treated TNBC cells with 0 or 5.5 × 10^9^ PEG-NSs/mL and/or 0 or 0.25 μM Pd[DMBil1]-PEG_750_ for 24 h. The cells were then irradiated with the 808 nm laser with 0.75 W/cm^2^ output power for 2 min/well to activate PTT and/or with λ_exc_ > 500 nm light on the light plate for 10 min to activate PDT. Following overnight incubation, cellular metabolic activity was assessed using Alamar Blue cell viability assays. Cells treated with PEG-NSs and/or the Pd[DMBil1]-PEG_750_ PSs without light irradiation experienced minimal losses in metabolic activity, demonstrating that neither agent alone or in combination is toxic without the appropriate light treatment (Figure 5). Further, we found that cells treated with PEG-NSs and the 808 nm laser, as well as cells treated with both agents and only 808 nm light, experienced 11% and 15% decreases in metabolic activity, respectively, compared to cells treated with no light. Additionally, cells treated with either Pd[DMBil1]-PEG_750_ or both agents and only exposed to the λ_exc_ > 500 nm light plate experienced 45% losses in viability. These results demonstrate that PTT and PDT can impact cell viability independently and with minimal cross-over between the two light sources, as cells treated with PEG-NSs and the λ_exc_ > 500 nm light plate or cells treated with Pd[DMBil1]-PEG_750_ and the 808 nm laser experienced only minimal loss of cell viability. 

Interestingly, cells co-treated with both Pd[DMBil1]-PEG_750_ and PEG-NSs that were exposed to both light sources experienced a 67% loss of metabolic activity, which is substantially better than either therapy alone (Figure 5). To assess if this effect is additive or synergistic, we calculated the coefficient of drug interactions (CDI). In this equation, a CDI > 1 indicates additive effects, whereas a CDI < 1 indicates synergistic effects. Using cell viability from cells treated with both PEG-NSs and Pd[DMBil1]-PEG_750_ and each mode of irradiation, we calculated a CDI = 0.7, demonstrating that PTT and PDT work synergistically to induce cell death.

**Figure 6:**
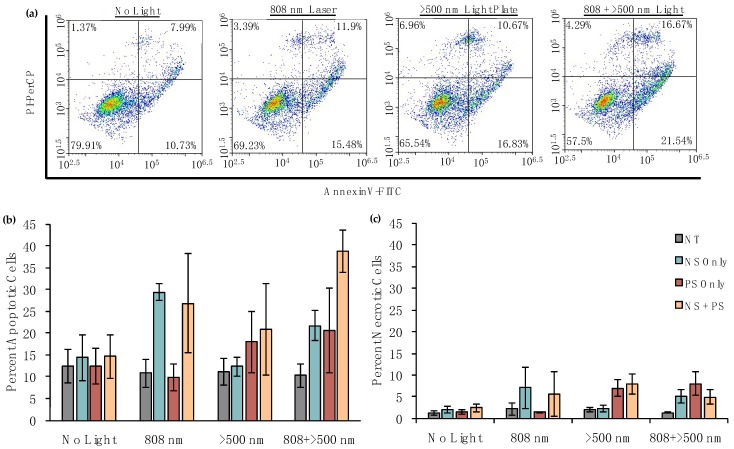
Analysis of the mechanism of cell death using AnnexinV/PI assays following treatment with PEG-NSs and/or Pd[DMBil1]-PEG_750_ photosensitizers (PSs) and each light source. (**a**) Representative scatter plots showing the percentage of cells undergoing apoptosis versus necrosis following treatment with Pd[DMBil1]-PEG_750_ and PEG-NSs with each light source; (**b**) Percentage of apoptotic cells; and (**c**) Percentage of necrotic cells following treatment. Data shown are compiled from four independent experiments.

After showing that dual PTT/PDT is a highly effective method for inducing cell death and gives better results than either therapy alone, we next evaluated the mechanism by which phototriggered cell death proceeds. This is important for any phototherapy because apoptosis or necrosis result in extremely different responses in the tumor microenvironment, so it is key to evaluate the mechanism of cell death in vitro. It is ideal to induce apoptosis rather than necrosis because the latter causes the release of intracellular components that lead to local inflammation and can stimulate growth of secondary tumors in distant sites [9,11]. Alternatively, apoptosis is anti-inflammatory and therefore discourages disease progression [10]. To assess the mechanism of cell death, we treated MDA-MB-231 cells with 0 or 5.5 × 10^9^ PEG-NSs/mL and/or 0 or 4 μM Pd[DMBil1]-PEG_750_ overnight and then irradiated for 2 min/well with the 808 nm laser at 0.85 W/cm^2^ and/or 20 min with the λ_exc_ > 500 nm light plate. Following 1 h incubation, AnnexinV (FITC channel) and PI (PerCP channel) staining was performed and analyzed by flow cytometry. In these experiments, cells that stain positive for AnnexinV only are undergoing early apoptosis (bottom right quadrant), cells experiencing late apoptosis stain positive for both AnnexinV and PI (top right quadrant), and cells undergoing necrosis stain positive for only PI (top left quadrant) (representative scatter plots from each light source are shown in Figure 6a). Importantly, these results show that dual PTT/PDT induces primarily apoptotic cell death (quantified data in Figure 6b,c). Adding the percentage of cells undergoing either early or late apoptosis revealed that ~39% of cells treated with both photoresponsive materials and both light sources undergo apoptotic cell death, compared to less than ~5% undergoing necrosis under the conditions tested here. As such, of the cells that undergo phototriggered cell death, an overwhelming proportion (nearly 90%) die via an apoptotic pathway.

## 4. Discussion

The two photoresponsive materials used here, PEG-NSs and Pd[DMBil1]-PEG_750_ PSs, are ideal platforms for PTT and PDT, respectively. We previously demonstrated that the Pd[DMBil1]-PEG_750_ PSs are highly effective and safe for PDT in TNBC cells with a substantially higher phototoxicity index than existing PSs [28]. Further, we have previously utilized PEG-NSs and NSs coated with therapeutic agents (such as siRNA and antibodies) to treat cancer [4,44], and clinical trials have shown that PEG-NSs are safe to use in humans [25,26,27,45]. Our overarching goal in this work was to evaluate combined PTT/PDT using PEG-NSs and the Pd[DMBil1]-PEG_750_ PSs as a treatment strategy for solid tumor cancers that can overcome the limitations associated with each individual therapy and induce primarily apoptotic cell death.

PDT as a standalone therapy has several key limitations. First, most photosensitizers are activated by low wavelengths of light that cannot penetrate tissue to reach tumors. Further, photosensitizers require oxygen to be available in order to be activated, and the interior of solid tumors is highly hypoxic. Thus, any photosensitizers within these hypoxic regions cannot efficiently mediate production of ^1^O_2_ and PDT [13,19]. Alternatively, PTT using PEG-NSs that are activated with tissue-penetrating NIR light has been widely studied and does not depend on tumor oxygen levels, but the treatment parameters must be carefully tailored to induce apoptosis, rather than necrosis [9,46,47]. The need to mitigate photoinitiated necrotic cell death is significant because this mechanism leads to the release of damage associated molecular patterns (DAMPs) that can promote the formation of secondary tumors in distant sites [9]. Low-energy irradiation that results in mild hyperthermia, however, can promote apoptosis that is anti-inflammatory and that can lead to beneficial immune responses that improve tumor regression [9]. We hypothesized that dual PTT/PDT as a strategy to treat solid tumors could ensure that either heat or ^1^O_2_ is produced throughout the entire tumor space, and that the ensuing mechanism of cell death would primarily be apoptosis. A key advantage of this strategy is that it offers the high specificity afforded by both treatment types while also decreasing the potential for off-target effects to healthy tissues because low doses of each agent and its respective activating light source can be applied. Additionally, compared to strategies that directly attach photosensitizers to the surface of NPs [29], our strategy ensures that both the photosensitizers and PEG-NSs can penetrate the tumor space so that ^1^O_2_ or heat are generated throughout, although the precise intratumoral distribution of these agents remains to be investigated in future in vivo studies.

To evaluate dual PTT/PDT mediated by PEG-NSs and Pd[DMBil1]-PEG_750_ as a strategy for pro-apoptotic cancer therapy_,_ we investigated the agents’ photophysical properties and stability in cell culture medium, their ability to enter cells, and their ability to induce light-activated cell death more effectively when combined than when applied individually. Our results demonstrate that both materials remain as separate entities and are stable over a 24 h incubation period in cell culture medium, as their extinction spectra and the PEG-NSs’ hydrodynamic diameter do not change over this timeframe. These results confirm that combining the PEG-NSs and Pd[DMBil1]-PEG_750_ PSs does not alter the photophysical properties of each material, and therefore they maintain their abilities to mediate PTT or PDT. 

Our results also show that PEG-NSs and Pd[DMBil1]-PEG_750_ can bind and/or enter TNBC cells and that dual PTT/PDT mediated by these agents is more effective than PTT or PDT alone, as the resultant viability of cells treated with dual PTT/PDT is lower than the viability of cells treated with either individual therapy. Importantly, our studies also confirmed that dual PTT/PDT induces primarily apoptosis, which is vastly preferred over necrotic cell death for the reasons discussed above. We calculated a CDI for dual therapy of 0.7, which indicates that this treatment strategy is highly synergistic. This validates our hypothesis that mild hyperthermia induced by PTT and toxic ^1^O_2_ produced by PDT can be used to induce apoptotic cell death, and that the two therapies can be applied simultaneously for a more comprehensive treatment approach. 

Many previous approaches that evaluate dual PTT/PDT for cancer treatment have utilized PSs physically tethered to the surface of nanoparticles or embedded within the nanoparticle cores [29]. The benefits of this approach are that the nanoparticles act as the delivery vehicles for the PSs, and the nanoparticles, upon irradiation with tissue-penetrating NIR light, may upconvert the absorbed light to indirectly activate the attached PSs [33,34,48]. However, these nanoparticle-PS conjugates are still limited by spatial variations (such as hypoxic regions and uneven distribution) within tumors. In our approach, we combined PSs and nanoparticles into the same solution, but did not tether them together, with the hypothesis that both could accumulate within tumors independently so that heat and ^1^O_2_ can be produced throughout. Future studies will evaluate the biodistribution and intratumoral distribution of PEG-NSs and Pd[DMBil1]-PEG_750_ to validate this hypothesis. The results presented here demonstrate that the combined application of these materials for dual PTT/PDT has substantial potential as a synergistic and pro-apoptotic cancer therapy. A key advantage of PEG-NSs and Pd[DMBil1]-PEG_750_ as photoresponsive therapeutics is that they display high safety profiles and high efficiencies for PTT or PDT, making their combination a viable therapeutic strategy. Thus, our therapeutic approach serves as a basis for future investigation into the use of these nanomaterials to deliver and work synergistically to treat solid tumors. 

## 5. Conclusions

In this study, we evaluated dual PTT/PDT with PEG-NSs and the biladiene-based PS, Pd[DMBil1]-PEG_750_, as a treatment strategy for TNBC, to overcome limitations associated with each individual therapy. Through these experiments, we demonstrated that dual therapy is more effective than either therapy alone, and that their combination is synergistic. Accordingly, lower laser powers and treatment dosages may be utilized for dual PTT/PDT therapy than for either mono-therapy alone, which serves to enhance the safety and specificity of treatment by minimizing off-target effects and preferentially impacting diseased cells. We showed that this combination strategy overwhelmingly triggers apoptotic cell death, which is ideal for any phototherapy in order to minimize inflammation that can lead to disease recurrence. Dual PTT/PDT mediated by PEG-NSs and Pd[DMBil1]-PEG_750_, respectively, warrants further investigation as a strategy to treat TNBC and other solid tumor cancers.
